# Staged Non-simultaneous Bilateral Patellar Tendon Rupture Treated With Direct Repair and Suture Tape Augmentation

**DOI:** 10.7759/cureus.94275

**Published:** 2025-10-10

**Authors:** Yuko Yagi, Hironori Kojima, Aiichiro Yamamoto

**Affiliations:** 1 Orthopedics, Otakanomori Hospital, Kashiwa, JPN; 2 Orthopedic Surgery, The University of Tokyo, Tokyo, JPN

**Keywords:** augmentation, bilateral injury, diabetes mellitus, knee extensor mechanism, obesity, patellar tendon rupture, staged rupture, surgical repair, suture tape, tendon degeneration

## Abstract

Bilateral patellar tendon rupture is an exceedingly rare injury, with most reported cases involving simultaneous ruptures in patients with predisposing systemic diseases or medication use. This report describes a unique case of a staged, non-simultaneous bilateral patellar tendon rupture that was surgically managed.

A 47-year-old man with class I obesity (body mass index, 33 kg/m^2^) and undiagnosed diabetes mellitus, and a history of long-term basketball participation, sustained a right patellar tendon rupture after a workplace accident. The injury went unrecognized, and two weeks later, a second low-energy fall led to a rupture of the left patellar tendon. Physical examination revealed bilateral patella alta, and radiographs confirmed the diagnosis, with marked calcification noted in the right tendon consistent with chronic changes.

The patient underwent a successful direct repair of both patellar tendons, augmented with a suture tape. While both knees achieved favorable functional recovery at the nine-month follow-up, the subacute right knee showed slightly reduced range of motion and residual stiffness compared to the acute left knee.

This case highlights that staged bilateral ruptures can occur in patients with metabolic and mechanical risk factors. The slightly reduced function on the subacute side suggests that surgical repair performed within two weeks may yield more favorable outcomes than a delayed repair.

## Introduction

Patellar tendon rupture is an uncommon injury, with a reported incidence of 0.68 per 100,000 individuals [1]. It usually results from sudden eccentric quadriceps contraction with the knee in a semi-flexed position, although direct trauma can also be a cause [2, 3]. Bilateral knee extensor mechanism ruptures are considerably rarer than unilateral cases. They are typically associated with underlying predisposing factors, including obesity, systemic or connective tissue diseases, and the use of specific medications [4, 5]. In addition, chronic microtrauma related to repetitive overuse has also been implicated [6, 7].

Surgical treatment is generally required for patellar tendon rupture. Primary tendon repair is most often performed in the acute setting, sometimes augmented with reinforcement techniques. In contrast, tendon reconstruction is typically reserved for chronic cases [8, 9].

In our case, the initial unilateral rupture went unrecognized by the patient, and a subsequent fall two weeks later led to rupture of the contralateral side. To our knowledge, surgically managed cases of a staged, non-simultaneous bilateral patellar tendon rupture are exceedingly scarce. We describe this unique case of a 47-year-old man treated with direct tendon repair augmented by a suture tape, resulting in a favorable clinical outcome.

## Case presentation

A 47-year-old man presented to the emergency department with bilateral knee pain and an inability to ambulate following a second fall. The initial injury occurred in his right knee two weeks earlier. While at a construction site, he fell approximately 70 cm, and his right knee became entrapped in 90° flexion by a metal bar. He then sustained a direct blow to the knee from a falling object. This incident resulted in pain and instability in his right knee. He subsequently required a cane for ambulation but experienced recurrent episodes of knee instability, which led to an accidental fall on the street approximately two weeks later. During this second fall, he landed on both knees, after which his left knee also became unable to extend. The pain, which had been confined to his right knee, now became bilateral.

**Figure 1:**
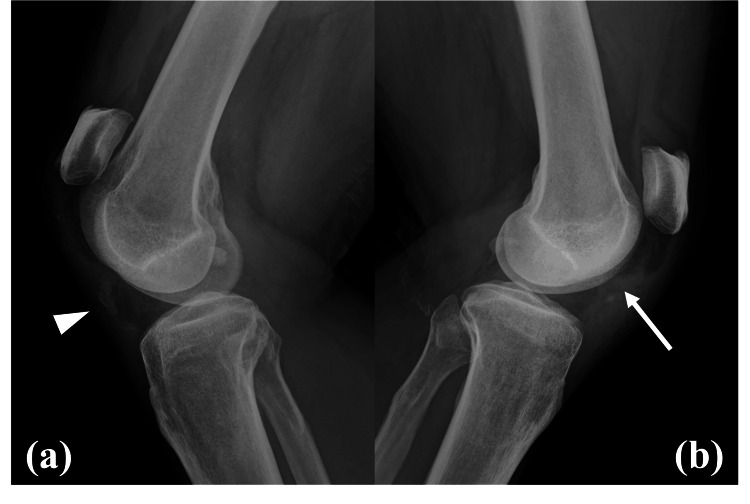
Preoperative lateral radiographs of the knees (a) right knee and (b) left knee. Both images demonstrate patella alta,  with increased density of the infrapatellar fat pad and loss of the sharp, linear margins of the patellar tendon. The arrowhead indicates marked calcification within the right patellar tendon, consistent with chronic degenerative changes, and the arrow indicates increased density of the infrapatellar fat pad.

On physical examination, palpable defects were noted at the distal patella, and he was unable to perform a straight leg raise bilaterally. Radiographic evaluation revealed bilateral patella alta, with Insall-Salvati ratios of 2.42 on the right and 1.98 on the left (Figure 1). The Insall-Salvati ratio is defined as the ratio of patellar tendon length to patellar length, and values greater than 1.2 are indicative of patella alta [10]. In addition, radiographs demonstrated increased density of the infrapatellar fat pad, as well as loss of the sharp, linear margins of the patellar tendon. Marked calcification was also noted within the right patellar tendon, consistent with chronic changes. These findings confirmed the diagnosis of bilateral patellar tendon rupture. 

The patient had no known history of systemic disease and denied prior use of corticosteroids or fluoroquinolones. He had played basketball through high school but reported no previous history of knee pain or frequent falls. He presented with class I obesity (body mass index, 33 kg/m^2^). Laboratory testing on admission revealed previously undiagnosed diabetes mellitus, with an HbA1c of 7.5% (Table 1). Screening for other potential systemic risk factors, including connective tissue disease, yielded no significant findings. Surgical repair was planned nine days after admission.

**Table 1:** Laboratory findings WBC: white blood cell count; RBC: red blood cell count; Hb: hemoglobin; Ht: hematocrit; Plt: platelet count; TP: total protein; Alb: albumin; CK: creatine kinase; AST: aspartate aminotransferase; ALT: alanine aminotransferase; LDH: lactate dehydrogenase; ALP: alkaline phosphatase; γ-GTP: gamma-glutamyl transpeptidase; UA: uric acid; BUN: blood urea nitrogen; Na: sodium; K: potassium; Cl: chloride; ANA: anti-nuclear antibody; dsDNA: double-stranded DNA; SSA: Sjögren’s-syndrome-related antigen A; SSB: Sjögren’s-syndrome-related antigen B; RF: rheumatoid factor; CCP: cyclic citrullinated peptide; PTH: parathyroid hormone; TSH: thyroid-stimulating hormone; FT3: free triiodothyronine; FT4: free thyroxine.

	Test	Result	
Hematology	WBC	12860	/uL
	RBC	5.74	×10^6^/uL
	Hb	16.7	g/dL
	Ht	50.2	%
	Plt	268	×10^3^/μL
Biochemistry	TP	6.7	g/dL
	Alb	4.0	g/dL
	CK	143	U/L
	AST	30	U/L
	ALT	46	U/L
	LDH	227	U/L
	ALP	105	U/L
	γ-GTP	37	U/L
	UA	5.1	mg/dL
	BUN	13.2	mg/dL
	Creatinine	0.88	mg/dL
	Na	140	mEq/L
	K	3.3	mEq/L
	Cl	104	mEq/L
	HbA1c	7.5	%
Immunology	ANA	negative	
	Anti-dsDNA ab	negative	
	Anti-SSA ab	negative	
	Anti-SSB ab	negative	
	RF	17	IU/mL
	Anti-CCP ab	negative	
Endocrinology	Intact PTH	40	pg/mL
	TSH	0.505	μIU/mL
	FT3	2.77	pg/mL
	FT4	1.39	ng/dL

**Figure 2:**
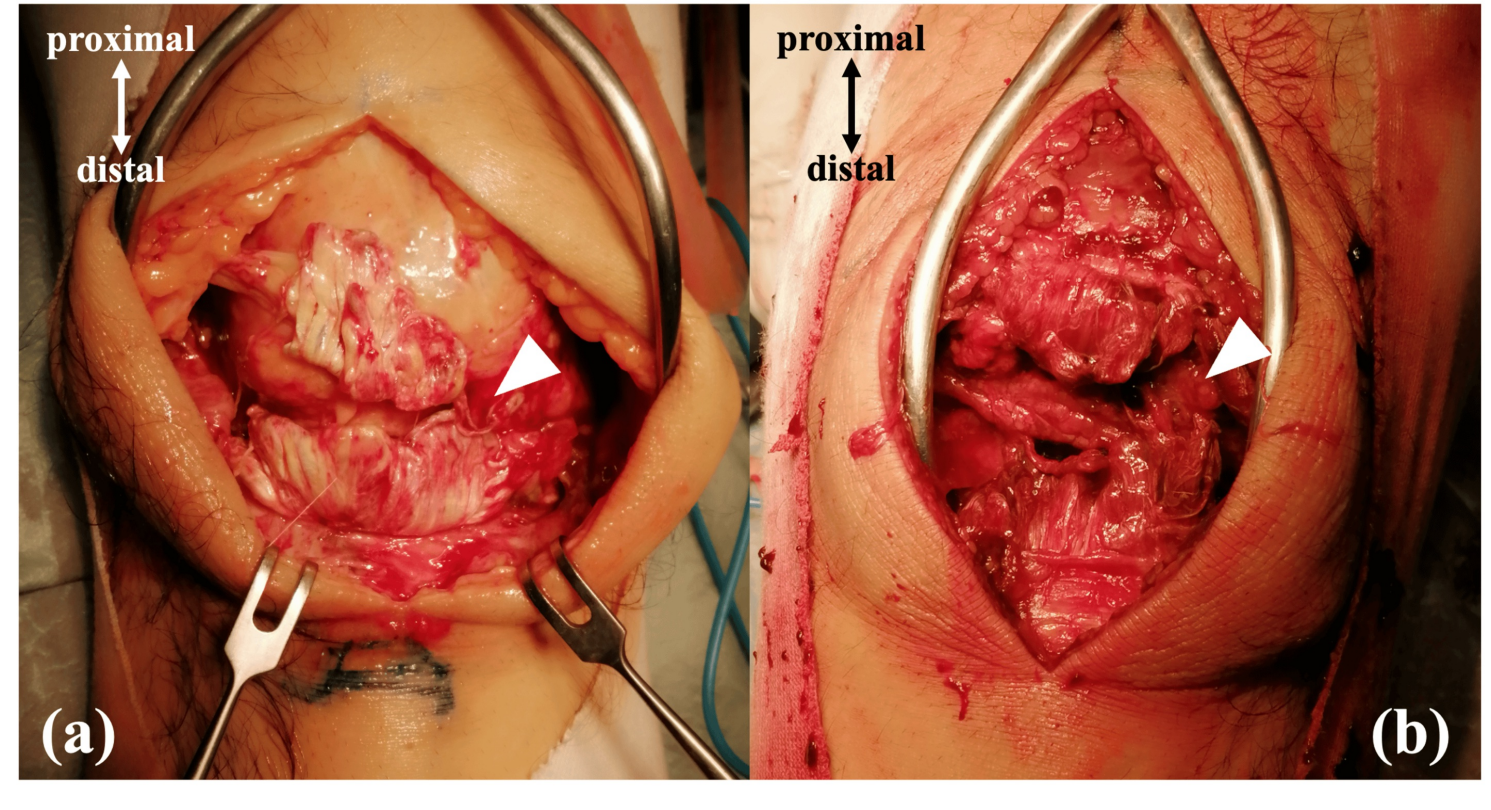
Intraoperative photographs (a) right knee and (b) left knee. Bilateral ruptures of the patellar tendon at their insertion on the inferior pole of the patella were observed (arrowheads). The right side showed whitish, scarred tissue, consistent with a subacute rupture.

The procedure was performed under general anesthesia with the patient in the supine position. A midline longitudinal skin incision was made, extending from the superior pole of the patella to the tibial tuberosity. On both sides, a rupture of the patellar tendon was confirmed at its insertion on the inferior pole of the patella (Figure 2). The right side showed an absorbed hematoma and whitish, scarred tissue, consistent with a subacute rupture. In contrast, the left side contained a large hematoma consistent with an acute rupture. The ruptured tendon ends were debrided to expose healthy tissue.

For primary repair, two No. 2 FiberWire sutures (Arthrex, Inc., Naples, Florida, USA) were placed in the patellar tendon using the Krackow technique. Three transosseous tunnels were drilled through the patella from the inferior to the superior pole. The sutures were passed through the tunnels and tied proximally, applying appropriate tension to restore patellar height. To augment the repair, a BroadBand tape (Zimmer Biomet, Inc., Warsaw, Indiana, USA) was passed through a bone tunnel in the tibial tuberosity. The tape was then routed anterior to the patellar tendon in a figure-of-eight configuration and secured proximally beneath the quadriceps tendon, thereby reinforcing the repair construct. The tendon ends were further approximated with 3-0 Polysorb sutures (Medtronic, Inc., Galway, Ireland), and the paratenon was closed. The wound was then closed in layers.

**Figure 3:**
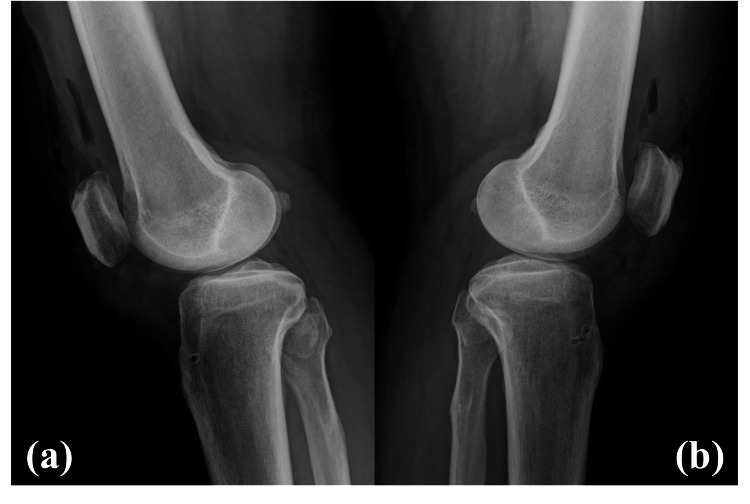
Postoperative lateral radiographs of the knees (a) right knee and (b) left knee. Both images demonstrate correction of patella alta after surgical repair.

Postoperative radiographs demonstrated successful reduction of patella alta, with Insall-Salvati ratios improved to 1.16 on the right and 1.10 on the left (Figure 3).

Following surgery, both knees were immobilized in splints. The patient began weight-bearing ambulation with knee braces one week postoperatively. Passive range-of-motion (ROM) exercises were initiated at two weeks, and the braces were discontinued at five weeks.

At the nine-month follow-up, he was pain-free and ambulating independently. Active ROM was −10° to 135° in the right knee and −5° to 145° in the left knee. According to the Lysholm Knee Scoring Scale, knee function was graded as good, with scores of 86 on the right and 91 on the left [11].

## Discussion

Bilateral patellar tendon rupture is exceedingly rare, and most cases involve simultaneous ruptures that occur in the context of tendon vulnerability due to specific risk factors [4, 5]. To date, there have been few reports of surgically managed staged, non-simultaneous bilateral patellar tendon rupture. We describe a case in which the initial rupture was unrecognized and subsequently followed by the contralateral rupture.

Predisposing systemic conditions that have been associated with tendon rupture include obesity, diabetes mellitus, systemic lupus erythematosus, chronic kidney disease, and other connective tissue disorders [5, 12-15]. Medication use, including corticosteroids (local or systemic) and fluoroquinolones, as well as chronic overuse injuries such as jumper’s knee, has also been reported as a risk factor [6, 7, 13, 16, 17]. Several predisposing factors may have contributed to this patient’s injury. He was obese and had previously undiagnosed diabetes mellitus, both of which are known to impair tendon quality by altering collagen structure and reducing tensile strength [18, 19]. Additionally, he had a history of long-term participation in basketball. Although he denied chronic knee pain, radiographs demonstrated marked calcification within the right patellar tendon, suggestive of degenerative changes related to chronic microtrauma [6, 7]. On the left side, calcification was less pronounced, yet rupture occurred after relatively low-energy trauma, raising the possibility of similar chronic tendon degeneration. No other established risk factors were identified in this case. Taken together, his athletic background and metabolic conditions likely created a weakened tendon environment that predisposed both knees to rupture-even under relatively low-energy circumstances, as in the second injury.

Both acute and subacute patellar tendon ruptures are generally managed surgically. Direct tendon repair is the most performed procedure, sometimes augmented with reinforcement techniques such as cerclage wiring [3,13]. Reconstruction using autografts or allografts may be necessary, especially in chronic ruptures (commonly defined as more than six weeks after injury), where tendon retraction and degeneration often preclude end-to-end repair [8, 9, 20]. However, despite these clinical observations, there is still no consensus or high-quality evidence regarding the optimal timing of surgery or the most appropriate surgical strategy depending on the stage of rupture. In the present case, direct repair was feasible bilaterally, and augmentation with a suture tape was performed to reinforce the construct. The subacute rupture on the right side (23 days after injury) could also be directly repaired, and restoration of patellar height and satisfactory stability were achieved bilaterally. Functional recovery was generally favorable; however, the patient reported slight residual stiffness in the right knee compared to the left, with some difficulty achieving a full squat. This subtle difference was most likely related to the subacute nature of the rupture. Additionally, the more pronounced radiographic calcification on the right side suggests greater underlying tendon degeneration, which may have also contributed to the poorer functional outcome.

Although conclusions cannot be drawn from a single case, the present findings suggest that surgical repair performed within two weeks may yield more favorable results than repair delayed to four weeks, raising the possibility that earlier intervention could optimize outcomes. Further investigation is warranted to determine whether the timing of repair, in combination with the degree of pre-existing tendon degeneration, influences long-term function in patellar tendon rupture.

This report has several limitations. Although radiographic patellar tendon calcification strongly suggested chronic tendon degeneration, histopathological confirmation was not obtained. Additionally, the nine-month follow-up period is relatively short to assess long-term functional outcomes. Moreover, the study lacks comparative data with other augmentation methods, such as autograft or allograft reconstruction, or with non-augmented direct repairs. Furthermore, it only includes the Lysholm Knee Score as a functional measure, which limits the assessment of postoperative activity levels and strength recovery.

## Conclusions

We report a rare case of staged, non-simultaneous bilateral patellar tendon rupture, managed surgically in a patient with obesity, diabetes mellitus, and probable chronic tendon degeneration. Both the acute and the subacute ruptures were treated with direct tendon repair augmented by a suture tape, resulting in a favorable overall recovery.

The slightly reduced ROM on the subacute side with radiographic tendon calcification suggests that the timing of surgical repair-as early as possible, ideally within two weeks-together with the extent of pre-existing tendon degeneration, may influence outcomes. This finding warrants further investigation to clarify how acute, subacute, and chronic stages of patellar tendon rupture impact the choice of surgical strategy and long-term functional results.
